# Simple DNA extraction for museum beetle specimens to unlock genetic data from historical collections

**DOI:** 10.1016/j.mex.2025.103236

**Published:** 2025-02-28

**Authors:** Hathal M. Al-Dhafer, Raju Balaji, Mahmoud S. Abdel-Dayem, Iftekhar Rasool, Amr Mohamed, Senthilkumar Palanisamy

**Affiliations:** a Plant Protection Department, College of Food and Agriculture Sciences, King Saud University, Riyadh, Saudi Arabia; b Center for Global Health Research, Saveetha Medical College and Hospital, Saveetha Institute of Medical and Technical Sciences, Thandalam, Kancheepuram District, Tamil Nadu, India; c Department of Entomology, Faculty of Science, Cairo University, Giza, Egypt; d Department of Genetic Engineering, SRM Institute of Science and Technology, Kattankulathur, Tamil Nadu, 603203, India

**Keywords:** Museum beetle, DNA extraction, CTAB method, PCR amplification, Next-generation sequencing, DNA extraction and PCR amplification of COI barcode

## Abstract

Museum beetle specimens are valuable resources for genetic analyses; however, obtaining DNA from aged specimens remains challenging due to degradation, desiccation, and contamination. In this study, we present a simple, low-cost protocol for extracting DNA from museum beetles, optimized using cetyltrimethylammonium bromide (CTAB). This method effectively addresses common issues such as DNA fragmentation and contamination, enabling the recovery of DNA suitable for downstream applications such as PCR and next-generation sequencing. It provides a reproducible, non-destructive approach to extracting genetic material from fragile beetle specimens, thereby facilitating molecular investigations in fields such as taxonomy and conservation biology. The protocol is summarized as follows:

• A method for DNA extraction is optimized for museum beetle specimens preserved for over 45 years.
• The protocol is non-destructive and compatible with PCR and next-generation sequencing.
• Multiple extractions can be pooled to increase yields, particularly when DNA concentrations are low.

This method broadens the possibilities for genetic analysis of historical specimens, offering new insights into long-term ecological and evolutionary processes.

## Graphical abstract

**Figure:**
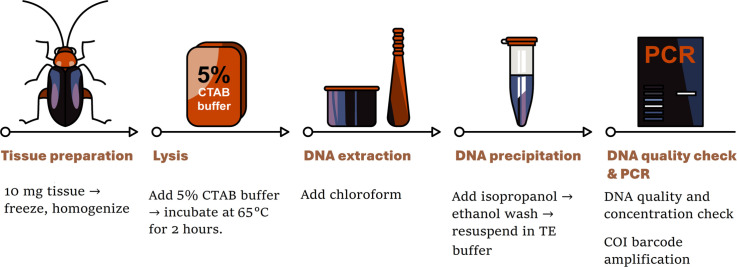

Specifications table

**Table:** 

Subject area:	Biochemistry, Genetics and Molecular Biology
More specific subject area:	DNA barcoding of museum beetles
Name of your method:	DNA extraction and PCR amplification of COI barcode
Name and reference of original method:	Huang, W., Xie, X., Liang, X., Wang, X., Chen, X., Effects of different pretreatments of DNA extraction from dried specimens of ladybird beetles (Coleoptera: Coccinellidae), Insects 10 (2019) 91. https://doi.org/10.3390/insects10040091
	Smith, A.D., Kamiński, M.J., Kanda, K., Sweet, A.D., Betancourt, J.L., Holmgren, C.A., Hempel, E., Alberti, F., Hofreiter, M., Recovery and analysis of ancient beetle DNA from subfossil packrat middens using high-throughput sequencing, Sci. Rep. 11 (2021) 12,635. https://doi.org/10.1038/s41598-021-91896-8
Resource availability:	Results of DNA extraction and PCR amplification of COI barcode from museum beetle were available within the article as gel picture

## Background

The order Coleoptera, or beetles, represents the most speciose group of animals, with over 400,000 described species [1]. Leaf beetles (Coleoptera: Chrysomelidae) is a diverse family of phytophagous insects with significant ecological importance [2]. Leaf beetles are one of the most extensively curated groups in museum collections from all parts of the world [2]. These specimens are invaluable for the study of historical biodiversity, taxonomy, and evolutionary processes. However, DNA quality degrades over time, and it has become difficult to obtain high-quality DNA from museum specimens [3,4]. Beetles stored in museums—primarily those gathered decades ago or ca. 45 years ago—undergo a variety of preservative methods that expose them to chemicals and environmental changes in temperature and humidity. These conditions highly contribute to DNA degradation. For instance, desiccation leads to fragmentation and loss of genetic material [5]. Therefore, conventional methods of DNA extraction usually result in the partial or even complete destruction of the specimen. This may be inappropriate if the materials are rare or critical, as in the case of type specimens. The main problems with extracting DNA from aged museum beetles represent extensive fragmentation and degradation of the genetic material [6]. Besides that, the extraction is complicated by the exposure to fluctuating preservation conditions and possible contamination either by modern DNA or chemical preservatives. Traditional methods used in DNA extraction usually work when the samples are fresh or well-preserved; in instances of degraded material, such methods may be applied ineffectively [7]. This problem is unpleasant in those cases where the morphological integrity of the specimen has to be preserved, as traditional methods may cause irreparable damage. However, despite these difficulties, DNA retrieval from beetle remains of older ages has great scientific importance. It allows genetic investigations into biological traces for the examination of historical biodiversity patterns, the determination of temporal changes in the genetic diversities of species, and the validation of historical taxonomic identifications. This genetic information will also be of importance in different conservation efforts that will help give a baseline against which current population conditions should be compared [7]. Recent advances in DNA extraction and sequencing technologies further improved the recovery of usable genetic material from museum collections and degraded specimens [8,9]. While several methods have been developed for DNA extraction from museum beetle specimens, each technique has particular advantages and disadvantages regarding its successful application [10,11]. Modified protocols addressing DNA fragmentation and contamination enabled the development of DNA extractions of good enough quality for molecular studies. These advantages open further perspectives for research into taxonomy, phylogenetics, and conservation biology. This study aims at designing and validating an optimized, cost-effective CTAB-based DNA extraction protocol adapted to aged beetle museum specimens. Key steps such as rapid freezing tissues in liquid nitrogen, using a potent lysis buffer, and incorporating RNase and Proteinase K ensure efficient recovery of intact DNA fragments. Furthermore, careful handling to avoid contamination and the ability to pool multiple extractions enhance DNA yield and purity, even in aged specimens. These features address the common issues of extensive fragmentation and contamination, enabling reliable downstream molecular applications such as PCR and sequencing. This will further increase the prospects for molecular studies of museum beetle specimens and will go a long way to help in taxonomy, phylogenetics, and conservation biology.

## Method details

### Nature of the specimens—Aged museum beetle sample

**Fig. 1:**
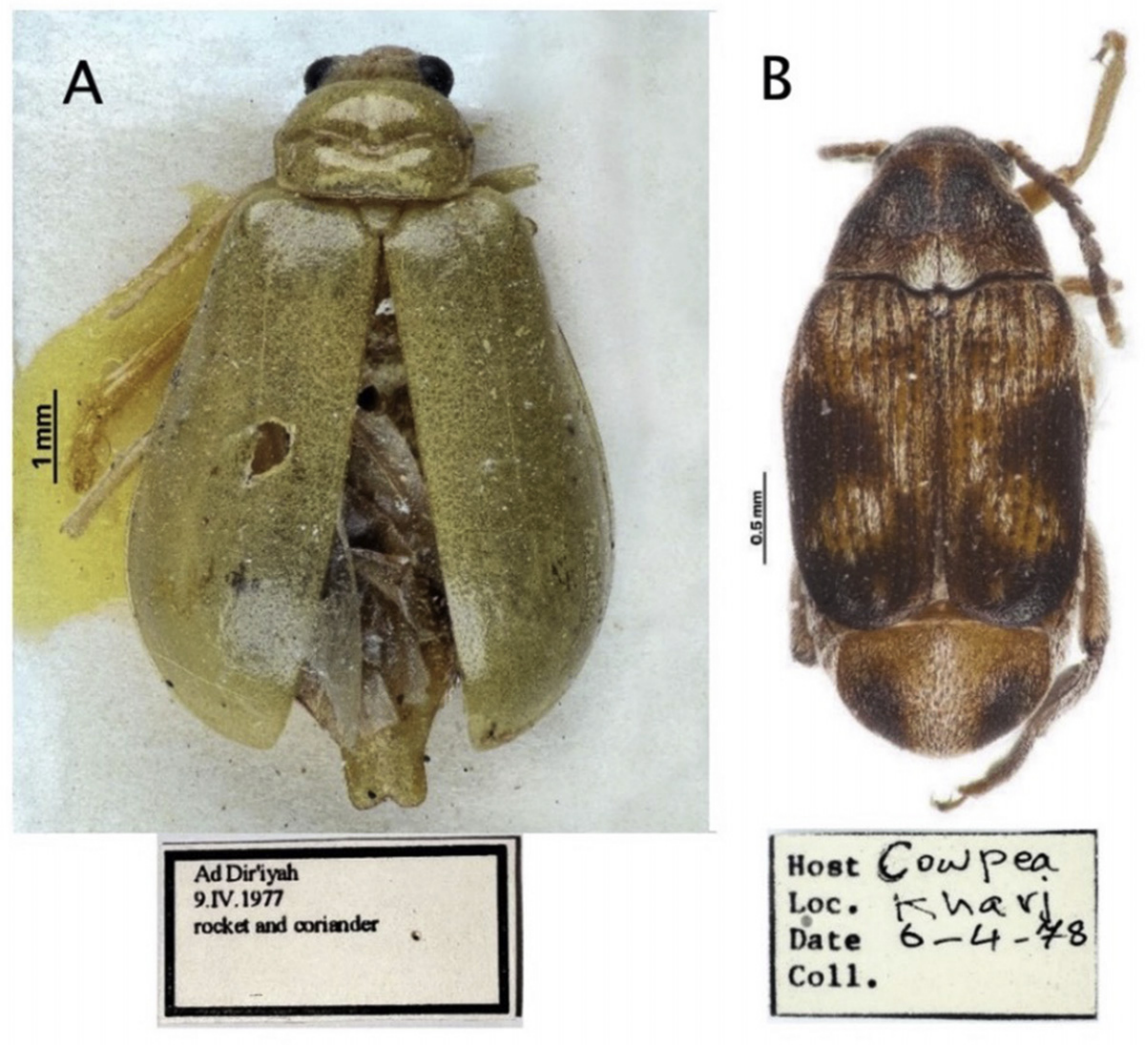
Representative images of museum beetle specimens used in this study. *Aulacophora foveicollis* (Lucas, 1849) (Coleoptera: Chrysomelidae: Galerucinae) is on the left (A). *Callosobruchus phaseoli* (Gyllenhal, 1833) (Coleoptera: Chrysomelidae: Bruchinae) is on the right (B).

The beetle specimens used in this study were obtained from natural history museums, where they had been preserved for 45–48 years (Fig. 1). These samples were stored under various environmental conditions that contributed to DNA degradation, including fluctuations in temperature, preservatives, and desiccation. To minimize morphological damage and maintain specimen integrity for further research, tissue samples were taken from less disturbed areas of the beetles, such as the legs or elytra. Small tissue samples, ranging from 5 to 10 mg, were carefully excised using sterile scalpels and tweezers to minimize contamination. The samples were then stored at −20°C to prevent further degradation until the DNA extraction process began. Strict sterile techniques and specialized equipment were employed throughout to avoid contamination from modern sources of DNA. Although these museum specimens present challenges due to their highly fragmented DNA and extended preservation period, they remain valuable resources for molecular studies, including DNA barcoding, phylogenetic analysis, and species identification.

## Materials

The lysis buffer in this protocol consists of 5 % CTAB, 100 mM Tris–HCl (pH 8.0), 20 mM EDTA, 1.4 M NaCl, and 0.2 % β-mercaptoethanol. Chloroform, cold isopropanol, and 70 % ethanol are required for the extraction process. A TE buffer, prepared with 10 mM Tris–HCl and 1 mM EDTA at pH 8.0, is also needed. Additional optional reagents include Proteinase K (20 mg/mL) and RNase A (10 mg/mL). The sterile tools required for the procedure are scalpels, tweezers, and pipette tips. The protocol also requires a mortar and pestle or bead mill homogenizer, along with 1.5 or 2.0 mL microcentrifuge tubes. An incubator or heat block, as well as liquid nitrogen, are also necessary. **Safety precautions** include wearing gloves, a lab coat, and safety goggles when handling chemicals.

### Reagents and solutions

**Fig. 2:**
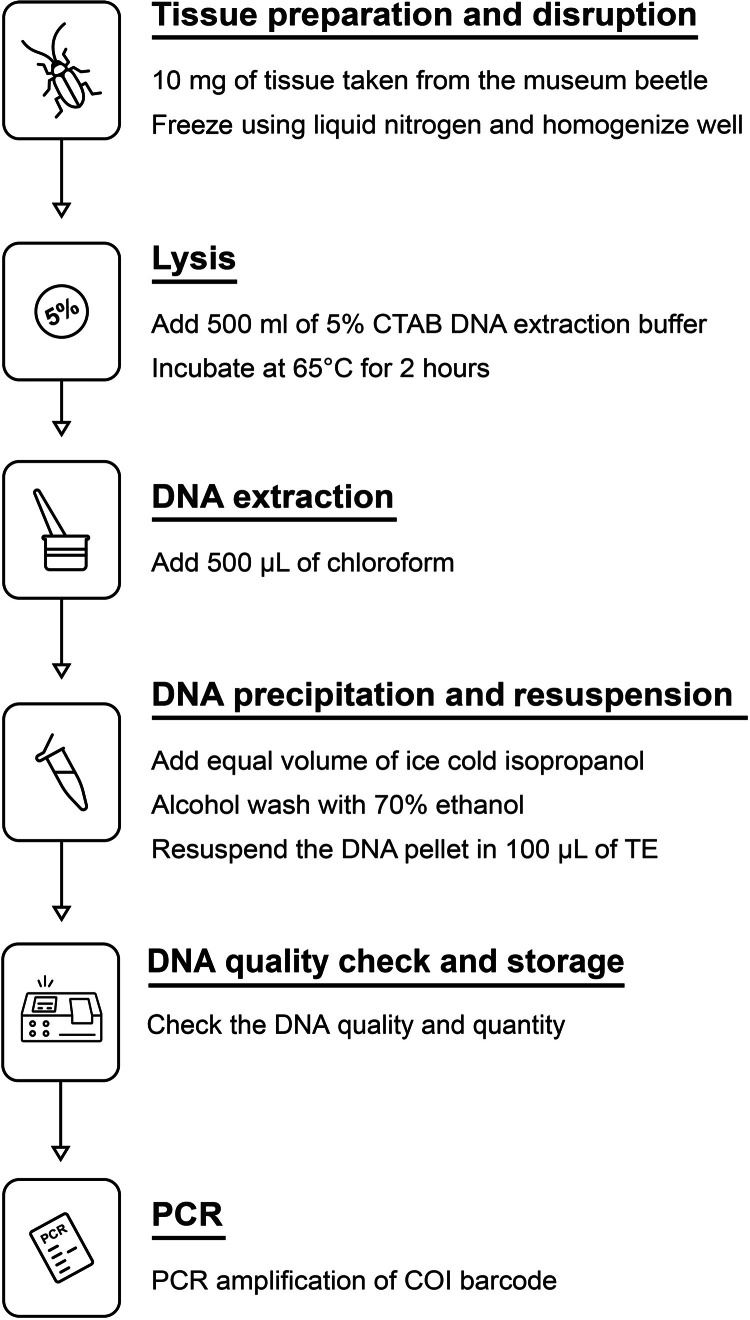
Procedure for DNA extraction from the museum beetle specimens.

The DNA was extracted, and the COI barcode was PCR-amplified from seven beetle specimens that had been preserved for >40–45 years. Specimens with variable storage conditions were chosen from less-handled areas like the legs and thoraces from the museum collection of King Saud University Museum of Arthropods (KSMA), Plant Protection Department, College of Food and Agriculture, King Saud University, Saudi Arabia. Appropriate sterile techniques were maintained at all times; wherever possible, tools and surfaces were sterilized to minimize the opportunity for contamination. A schematic methodology is outlined in detail in the flow diagram depicted in Fig. 2.

## DNA extraction protocol

#### Tissue preparation and disruption

1. Excise tissue samples from the selected beetle parts, about 5–10 mg, using sterile scalpels and tweezers.
2. Transfer tissues into sterile microcentrifuge tubes.
3. Rapid freeze the tissues in liquid nitrogen.
4. Conduct mechanical rupture of tissues using a sterile mortar and pestle.

#### Lysis

5. Incubate with 500 µL of preheated CTAB lysis buffer (65°C).
6. Add 2 µL of RNase A at 10 mg/mL concentrations to remove RNA.
7. Add 25 µL of proteinase K (20 mg/mL) to digest proteins.
8. Incubate tubes at 65°C for 2 h. Mix gently every 15 mins for enhanced lysis; in the case of heavily degraded samples, incubation should be extended overnight.

#### DNA extraction

9. Add 500 µL of chloroform to the lysates.
10. Gently invert the tubes for mixing for about 10 mins.
11. Centrifuge at 12,000 rpm for 15 mins at room temperature.
12. Carefully transfer the aqueous phase containing DNA into a new microcentrifuge tube.

#### DNA precipitation and resuspension

13. Add cold isopropanol to the aqueous phase in equal volume.
14. Incubate at −20°C for 1 hour to precipitate the DNA.
15. Pellet the DNA by centrifugation at 12,000 rpm for 10 min at 4°C.
16. Wash the DNA pellet twice with 70 % ethanol and once with 100 % ethanol to remove salts.
17. Allow DNA pellets to air-dry for 10 mins to avoid over-drying.
18. Resuspend the DNA pellet in 100 µL of TE buffer (10 mM Tris–HCl, 1 mM EDTA, pH 8.0).
19. Incubate at 37°C for 30 min to ensure complete resuspension of the DNA pellet.

#### DNA quality check and storage

20. The quality and quantity of the extracted DNA should be checked by running 2 µL of each sample on a 1 % agarose gel.
21. Spectrophotometric analysis is done to confirm the DNA concentration and purity by the A260/A280 ratio.
22. DNA can be stored at −20°C.

## Method validation

The improved CTAB protocol was validated based on the quality and yield of DNA extracted from beetle specimens that were aged for over ∼45 years.

### DNA integrity

DNA integrity was assessed using agarose gel electrophoresis.

**Fig. 3:**
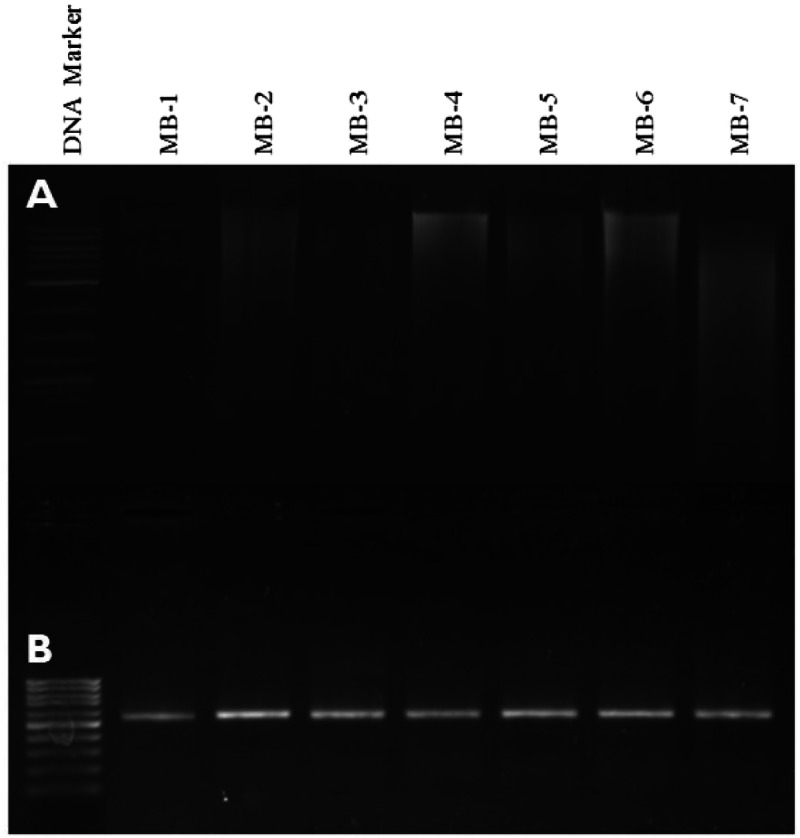
Agarose gel electrophoresis of the genomic DNA from museum beetle samples. Lanes 2–8 (Top Gel: 3A) and the PCR-amplified COI barcode marker (650 bp) (Bottom gel: 3B) from the respective samples, along with Lane 1: 1 kb DNA ladder (Top gel) and 100 bp DNA ladder (Bottom gel), were marked as M.

Clear bands with minor smearing were observed, indicating natural degradation over time (Fig. 3A).

### Spectrophotometric analysis

Spectrophotometric analysis revealed A260/A280 ratios between 1.7 and 1.9.

This suggests that the DNA obtained had high purity, despite the age and conditions of the samples.

### DNA yield

DNA yields ranged from 5 to 50 ng/µL, depending on specimen preservation.

On average, DNA recovery was 2000 ng per sample.

### PCR amplification

Extracted DNA was subjected to PCR targeting a 650 bp region of the mitochondrial cytochrome c oxidase I (COI) gene.

Universal primer pairs used:

• LCO1490 5′-GTCAACAAATCATAAAGATATTGG-3′
• HCO2198 5′-TAAACTTCAGGGTGACCAAAAAATCA-3′ [12].

The PCR mixture (30 µL total) contained:

• 1 µL of template DNA (50 ng)
• 1 µL of each primer (5 pmol/µL)
• 1 µL of Taq DNA polymerase (1U/µL)
• 1 µL of dNTPs (10 mM)
• 3 µL of 10X Taq Buffer with MgCl₂
• Sterilized nuclease-free water to a final volume of 30 µL.

The PCR reaction cycles were as follows:

• Initial denaturation at 95°C for 5 mins
• 35 cycles of denaturation at 95°C for 30 ss
• Annealing at 55°C for 30 ss
• Extension at 72°C for 1 min
• Final extension at 72°C for 5 mins.

### PCR product analysis

PCR products were checked by electrophoresis on a 1.2 % agarose gel in 0.5x TBE buffer.

Successful amplification of the COI (650 bp) barcode marker was achieved.

Well-defined bands, corresponding to the expected amplicon sizes, were observed (Fig. 3B).

In well-preserved specimens, higher molecular weight bands confirmed that the protocol can recover intact DNA under good preservation conditions.

### In conclusion

The modified CTAB protocol successfully yielded DNA from aged museum beetle specimens.

The protocol provides enough DNA for further PCR and sequencing.

This method is low-cost and suitable for molecular studies on historical beetle specimens, including taxonomic, phylogenetic, and conservation analyses.

Fig. 3 depicts agarose gel electrophoresis results for genomic DNA extraction (A) and PCR-amplified COI barcode marker (B) from museum beetle samples.

• Panel A: Genomic DNA from seven museum beetle (MB) samples (lanes MB-1 to MB-7) is shown, with clear high-molecular-weight bands visible, indicating successful DNA extraction. Lane 1 contains a 1 kb DNA ladder for size reference.
• Panel B: PCR amplification of the COI barcode marker (650 bp) is presented for the same samples (lanes MB-1 to MB-7), showing uniform and sharp bands, confirming successful amplification. Lane 1 contains a 100 bp DNA ladder for reference.

### Key findings

• High-quality genomic DNA was successfully extracted from all seven museum beetle samples, as evidenced by distinct bands in Panel A.
• The COI barcode marker (650 bp) was successfully amplified for all samples, as shown by consistent and sharp bands in Panel B.
• The results validate the DNA extraction and amplification protocols for degraded museum beetle specimens.

## Limitations

The main limitations of this protocol include high DNA degradation and low yield, especially from heavily degraded specimens, which hinder the recovery of longer amplicons for more advanced genetic analyses. Preservation conditions among the specimens are inconsistent; hence, DNA quality could be affected. Contaminations are not completely avoided even with sterile techniques. No tests have been done on the effectiveness of this protocol with other insect species or beetles preserved in different methods. Future studies could benefit from adapting this protocol to other insect species and exploring different preservation methods used in museums, which would help increase its applicability across a wider range of specimens. Lastly, DNA fragmentation limits the amplification of larger or more complex regions. The third concern is the potential for minor morphological damage to specimens during the tissue excision process. Additionally, this method requires specialized equipment, such as liquid nitrogen, which may not be available in all laboratories.

## Concluding Remarks

DNA extraction from aged museum beetle specimens is often challenging due to degradation and contamination. In this study, we present a modified CTAB-based protocol that effectively addresses these two major issues by integrating both mechanical and chemical lysis techniques. The method is cost-effective and practical, providing a reliable approach to obtaining DNA from old specimens for molecular biology applications. The protocol enables the extraction of sufficient DNA for PCR amplification and sequencing, even from degraded samples. Our results demonstrate that high-quality DNA can be successfully obtained from preserved museum beetle specimens when handled carefully and following the established protocol. This work paves the way for meaningful genetic studies and analyses of historical specimens. Future research should focus on further refining the protocol and testing it on a broader range of specimens to enhance its robustness and versatility.

## Ethics statements

In accordance with institutional norms, we followed laboratory safety protocols and handled museum beetle specimens in a safe manner during our experimental investigations.

## CRediT authorship contribution statement

**Hathal M Al-Dhafer:** Conceptualization, Methodology, Software. **Raju Balaji:** Methodology, Validity tests, Data curation. **Mahmoud S Abdel-Dayem:** Conceptualization, Visualization, Investigation, Writing- Original draft preparation. **Iftekhar Rasool:** Methodology, Software, Validation. **Amr Mohamed:** Conceptualization, Methodology, Supervision, Writing- Original draft preparation, Writing- Reviewing and Editing. **Senthilkumar Palanisamy:** Conceptualization, Methodology, Software, Data curation, Writing- Original draft preparation, Writing- Reviewing and Editing.

## Declaration of competing interest

The authors declare that they have no known competing financial interests or personal relationships that could have appeared to influence the work reported in this paper.

## Data Availability

All data generated or analyzed during this study are included in this published article.
